# Two-Dimensional Black Phosphorus: Preparation, Passivation and Lithium-Ion Battery Applications

**DOI:** 10.3390/molecules27185845

**Published:** 2022-09-09

**Authors:** Hongda Li, Chenpu Li, Hao Zhao, Boran Tao, Guofu Wang

**Affiliations:** Liuzhou Key Laboratory for New Energy Vehicle Power Lithium Battery, School of Electronic Engineering, Guangxi University of Science and Technology, Liuzhou 545006, China

**Keywords:** two-dimensional black phosphorus, preparation, surface modification, lithium-ion battery applications

## Abstract

As a new type of single element direct-bandgap semiconductor, black phosphorus (BP) shows many excellent characteristics due to its unique two-dimensional (2D) structure, which has great potential in the fields of optoelectronics, biology, sensing, information, and so on. In recent years, a series of physical and chemical methods have been developed to modify the surface of 2D BP to inhibit its contact with water and oxygen and improve the stability and physical properties of 2D BP. By doping and coating other materials, the stability of BP applied in the anode of a lithium-ion battery was improved. In this work, the preparation, passivation, and lithium-ion battery applications of two-dimensional black phosphorus are summarized and reviewed. Firstly, a variety of BP preparation methods are summarized. Secondly, starting from the environmental instability of BP, different passivation technologies are compared. Thirdly, the applications of BP in energy storage are introduced, especially the application of BP-based materials in lithium-ion batteries. Finally, based on preparation, surface functionalization, and lithium-ion battery of 2D BP, the current research status and possible future development direction are put forward.

## 1. Introduction

With the development of a global economy and society, people’s demands for and use of energy are increasing day by day. The problems of environmental pollution and fossil energy shortage are becoming more and more obvious. For the sustainable development of human beings, it is urgent to seek the development of new energy and renewable resources. As a chemical energy storage device, lithium-ion batteries (LIBs) are widely used in portable electronic equipment [1], aerospace [2], military equipment [3], and electric vehicles [4] due to their advantages of high specific power, high energy density, long life, low self-discharge rate, and long storage time [5,6,7,8,9]. At present, LIBs have gradually replaced other batteries as the main power battery [10]. The development of smart grid and large-scale energy storage in recent years has put forward higher requirements on the energy density and power density of LIBs, which makes it particularly important to develop new LIBs with high energy density and power density.

Black phosphorus (BP) is the most stable of the three common allotropes of phosphorus (red phosphorus, white phosphorus, and BP) [11]. BP has four kinds of crystal structures: orthogonal, rhombus, simple cubic, and amorphous, and is an orthogonal crystal structure at room temperature and under pressure. BP has a layered structure like graphite, but the phosphorus atoms within the same layer are not in the same plane; it is a kind of honeycombed fold structure. There is a strong covalent bond in the layer, and a single electron pair remains, so each atom is saturated, and the atoms between layers act by van der Waals forces. Similar to graphite, the structure of BP allows the preparation of two-dimensional (2D) BP crystals. Some properties of 2D materials are not found in bulk materials. At present, graphene and 2D transition metal sulfide are pioneers in this field [12,13]. The performance of monolayer BP is superior to graphene and 2D transition metal sulfide mainly in semiconductor and photoelectric properties. Graphene has very high carrier mobility, but its zero-band gap effect makes it unable to realize semiconductor logic switch. Two-dimensional transition metal sulfide (e.g., MoTe_2_) has good semiconductor performance, and the transistor prepared by MoTe_2_ has good electrical regulation performance, but its low carrier mobility limits its application in the field of electronics [14,15]. As a new 2D material, 2D BP has become the star of the anode material for LIBs with adjustable band structure, excellent electrical properties, anisotropic mechanical, thermodynamic, and photoelectrical properties. Since SONY LIBs were commercialized in 1990, LIBs have become one of the research hotspots in the field of new energy due to its characteristics of high specific capacity, long cycle life, no pollution, and good safety.

Two-dimensional BP shows excellent mechanical, electrochemical, and thermodynamic properties, and has great research value in ultra-light materials, energy storage devices, flexible electronics, and other aspects [16,17,18,19,20]. BP was first synthesized by Bridgman [21] under high temperature and high pressure, but it was seldom studied due to the technical conditions at that time. In 2014, Zhang and Ye et al. [22] successfully obtained monolayer BP through the method of sellot-tape stripping, which promoted the further study of BP performance [23].

The emergence of 2D BP also promotes the development of a new energy field. With the progress of society and technology, the existing energy materials available to meet people’s needs are becoming less and less, which stimulates people’s continuous exploration in the field of new energy. LIBs, sodium ion batteries (SIBs), lithium sulfur batteries (LBS), magnesium ion batteries (MIBs), super capacitors, and other electrochemical energy storage devices are developing rapidly. In these energy storage devices, LIBs have been widely used. The 3C market (mobile phones, computers, cameras, etc.) has its most mature systems. However, LIBs are mostly confined to these small devices and have limited application in the power battery market. Therefore, researchers have been committed to exploring high-performance LIBs with high capacity, high speed, and long life. The electrode material determines the overall performance of LIBs. Most importantly, with a high theoretical specific capacity of 2596 mAh g^−1^, BP will shine in the field of energy storage [24]. Therefore, a large number of theoretical and experimental studies have been carried out on BP and BP-based electrode materials in order to seek new breakthroughs in the field of LIBs.

Given the advantages of BP in the field of energy storage, this paper summarizes and reviews the preparation, passivation, and lithium-ion battery applications of two-dimensional black phosphorus. Firstly, a variety of BP preparation methods are summarized. Secondly, starting from the environmental instability of BP, different passivation technologies are compared. Thirdly, the applications of BP in energy storage are introduced, especially the application of BP-based materials in lithium-ion batteries. Finally, based on preparation, surface functionalization, and lithium-ion battery of 2D BP, the current research status and possible future development directions are put forward.

## 2. Preparation of 2D BP

In 1914, Bridgman heated white phosphorus to 200 °C in an experiment and obtained BP crystals under a pressure of 1.2 GPa [25], but it did not get people’s attention for a long time. Not until 1981, when Maruyama et al. [26] dissolved white phosphorus in bismuth solution in a reaction kettle and kept it for 20 h at 400 °C. After slow cooling, BP with needle-like or rod-like structure was obtained, with a grain size of 5 × 0.1 × 0.07 mm^3^. The use of BP as an anode in LIB only gained attention in 2005. Due to its demanding synthesis method, a simple, efficient, and non-toxic preparation method that can be used in industrial production is extremely important for the development of BP.

The traditional preparation method of 2D BP (single layer or less layer) adopts scotch tape mechanical cleavage method from the bulk BP crystal, which has low preparation efficiency and low purity of 2D BP. Therefore, researchers conducted a large number of experiments to explore a variety of basic research preparation methods, including chemical vapor deposition (CVD) [27], exfoliation method [28], phase transition and solvothermal reaction method, and other methods.

### 2.1. Synthesis of Bulk BP

Mercury reflux is a method to reduce the pressure required for the preparation of BP. In 1955, Krebs et al. [29] first reported a method to prepare BP using metal mercury, which can be synthesized at the pressure of 3.5 × 10^7^~4.5 × 10^7^ Pa. BP can be prepared by mixing white phosphorus with metallic mercury and putting it into a pressure vessel and holding it at a certain temperature for several days. Due to the catalytic action of mercury, the activation energy required for the conversion of white phosphorus to BP is reduced, so the preparation pressure is also reduced. Although the mercury reflux method can produce BP under relatively mild conditions, metallic mercury is greatly harmful to the human body and the environment, it takes a long time to prepare, and it is necessary to remove metallic mercury from BP products in the later stage. Therefore, few researchers carry out relevant studies on it.

The bismuth melting method [26] is to prepare BP by dissolving white phosphorus in liquid bismuth and holding it at 400 °C for a long time (20–48 h). Since red phosphorus is insoluble in liquid bismuth and there is a certain risk in direct contact with white phosphorus, Mamoru Baba et al. [30] first heated red phosphorus as the precursor at a specific temperature to vaporize red phosphorus and condense it to produce white phosphorus. Meanwhile, they heated solid bismuth at 300 °C (melting point is 271.3 °C) to make it liquid. BP can be prepared after white phosphorus is dissolved in liquid bismuth at a high temperature and held for a period of time. The advantage of this preparation is the reduced risk of direct contact with white phosphorus. However, in general, the preparation process is relatively complex, the experimental process consumes a large amount of bismuth, the heat preservation time is longer, but also, the need to use strong acid bismuth from BP removal in the product will produce waste liquid in the process of environmental pollution. Thus, the bismuth melting method also was not developed well.

### 2.2. Exfoliation Methods

At present, the synthesis of single crystal BP usually uses the mineralizer method [27]. A certain proportion of Sn, SnI4, and RP are sealed in a vacuum quartz tube, and the single crystal is synthesized by setting a temperature gradient. In recent years, researchers have used different kinds of stripping methods to strip single crystal BP.

#### 2.2.1. Mechanical Exfoliation

**Figure 1:**
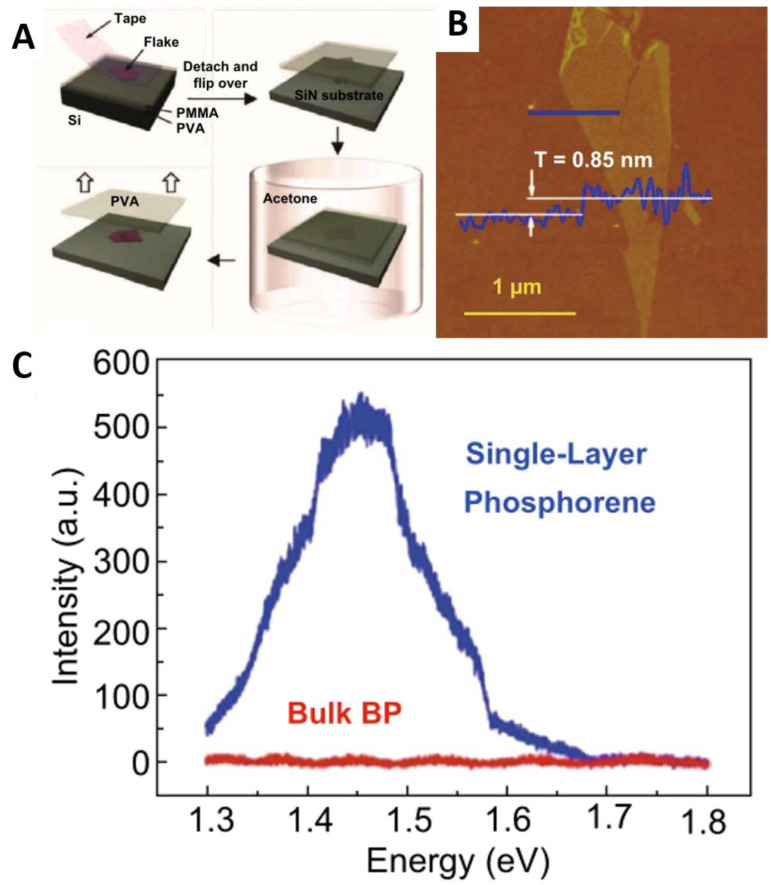
(**A**) Process of preparation of less layer BP by mechanical stripping [31]. (**B**) AFM image of BP prepared by mechanical stripping; (**C**) Photoluminescence spectrum. Reproduced with permission [22]. Copyright 2014, ACS Publications.

As BP has a similar layered structure to graphite, it can also be used to prepare BP film by referring to the method of mechanically peeling graphite to prepare graphene. The traditional mechanical stripping method is to peel BP repeatedly with transparent tape to achieve a few layers of BP film, as shown in Figure 1A [31]. Finally, with the clear tape residue, the final product is obtained [22,32,33]. Liu et al. [22] used this method to prepare phosphorus with different layers. As can be seen from the AFM image in Figure 1B, the thickness of single-layer phosphating film is 0.85 nm, larger than the theoretical calculation of 0.6 nm. The photoluminescence (PL) spectra (Figure 1C) shows that the energy gap of the monolayer phosphating film is 1.45 eV. In addition, the prepared BP chip with the thickness of 46 nm has a hole carrier mobility of 286 cm^2^ V^−1^ s^−1^. Guan et al. [34] improved the traditional mechanical stripping method of scotch tape. A layer of gold or silver about 10 nm was deposited on the Si/SiO_2_ substrate to enhance the adhesion between the BP body and the substrate, and then it was stripped. The phosphorescence prepared by the metal-assisted mechanical stripping method has a thickness of ~4 nm, a transverse size of ~50 μm, and a hole carrier mobility of 68.6 cm^2^ V^−1^ s^−1^. It can be seen that phosphorus obtained by the mechanical stripping method has an irregular size, uncontrollable layers, general electrical performance, and low efficiency, which can only be used for the study of infrastructure and performance [35,36,37,38].

#### 2.2.2. Sonication Liquid-Phase Exfoliation

Although the mechanical stripping method required relatively simple experimental conditions, it is labor intensive, time-consuming, has a low yield, and only a single form of phosphorusene can be prepared, so it is only suitable for basic laboratory characterization and research. In contrast, the ultrasonic stripping method can control the ultrasonic power to prepare different forms of BP nanoparticles, such as phosphorusene and BP quantum dots. Due to the advantages of low cost and easy operation, the method is often used for the preparation of nano-BP matrix composites. Brent et al. [39] first reported the study on the preparation of nanometer BP by ultrasonic stripping. They placed BP in N-methylpyrrolidone (NMP), controlled the bath temperature below 30 °C, and obtained BP nanosheets with a size of 200 × 200 nm and a thickness of 3.5–5 nm by continuous ultrasonic stripping for 24 h. Although the prepared nanosheets have high crystallinity, they have poor stability, a time-consuming preparation process, and low yield (less than 10%). In order to improve the stability of the nano-BP and stripping time, Halon et al. [40] used the solvent for N-cyclohexyl-2-pyrrolidone (CHP). After ultrasonic stripping for 5 h, they achieved a high quality as the multiple centrifugal supernatant fluid layer black phosphorus nanometer film, based on the solvation shell protection principle, and the stability of the preparation of nanometer BP were improved to a certain extent. In order to study the effect of centrifugal rate on the size and morphology of nano-BP, Late et al. [41] centrifuged BP at 3000, 5000, and 10,000 r/min, respectively, with NMP as solvent, and found that the increase of rotational speed was conducive to obtaining small and thin BP nanosheets.

In consideration of environmental friendliness, scale, and simplicity of preparation, Zhao et al. [42] prepared atomically thin BP nanosheets using ionic liquids instead of organic solvents for the first time, and also considered the stripping effect of nine ionic liquids. It was found that 1-hydroxyethyl-3-methyl imidazolium trifluoromethane sulfonate could be used as the stripping solvent to obtain dispersions of BP nanosheets with a concentration of 0.95 mg mL^−1^ (0.4 mg mL^−1^ in NMP), and the dispersions could stably exist in air for one month without obvious polymerization. The introduction of ionic liquid improves the stability and concentration of the dispersion, and is an ideal green stripping agent [43], but the yield of the preparation of BP nanoparticles is still not high, and the price of ionic liquid is more expensive. In order to improve the production rate of BP nanosheets and reduce the production cost of BP nanosheets, Su et al. [44] added an Li2SiF6 assisted intercalation water bath ultrasound for 5 h, and confirmed through comparative experiments that the production rate of BP nanosheets of Li2SiF6 assisted intercalation in DMSO was up to 75%. The BP nanosheets obtained by exfoliation have high purity and high crystallinity. The apparent monolayer thickness is (2.04 ± 0.18) nm, the average number of layers, and the transverse size are about 4 layers and 3.74 mm, respectively. In addition, ultrasonic stripping can prepare BP quantum dots as well as phosphorusene [45].

Although the sonication liquid-phase exfoliation method is simple to operate, not very time consuming, and has a high yield, a variety of organic solvents used will pollute the environment, and organic solvent molecules are easily adsorbed on the final products, affecting their inherent properties.

#### 2.2.3. Electrochemical Exfoliation

The electrochemical stripping method can regulate the voltage size in order to control the morphology of nano-BP, and has advantages of being a simple operation and having a low cost, and it can prepare BP, black phosphate quantum dots, and even the structure of the new nano-BP-perforated BP, 3D BP, a different morphology of nanometer BP. Thus, at present, it is a commonly used method for the preparation of BP.

Depending on the lamellar materials (BP) and the position (cathode, anode, and electrolyte), the electrochemical stripping method can be summarized as the anode stripping method [28], cathodic stripping method [46,47,48,49], or electrolytic stripping method [47]. These three kinds of stripping methods have a similar principle: in the selected electrolyte, the applied electric field of the generated gas or ions are inserted into the layer interface between the layers, thus it requires less stripping than even a single layer. Among them, the anode stripping method and electrolyte stripping method are more green and environmentally friendly (inorganic solution is mostly used as the stripping solvent), but the variety of nano-BP prepared is smaller and is easily oxidized, which is not conducive to the application of nano-BP. In contrast, the BP nanoparticles prepared by the cathode stripping method are rich in variety, and oxygen free radicals are not generated during the stripping process, so the BP nanoparticles prepared are relatively stable.

In the process of electrochemical stripping, the bulk BP acts as the working electrode and generates current by applying voltage. Due to the joint action of current and electrolyte on the layered structure, the bulk BP will be stripped into phosphorusene. The electrochemical stripping efficiency of BP depends on the selection of voltage and electrolyte. Erande et al. [28] used a massive BP crystal as anode, platinum wire as the counter electrode, 0.05 M Na_2_SO_4_ as electrolyte, and applied a voltage of +7 V, equivalent to 1 mA current. After 25 min of reaction, the solution turned light yellow, and after 90 min, the power was turned off. Finally, the solution was centrifuged to obtain atomic thin layer of phosphorusene, and the yield could reach more than 80%. However, the BP nanosheets after electrochemical stripping showed a wide thickness range (1.4–10 nm, corresponding to 3–15 layers). Due to the uneven size of the nanosheets, the BP mobility was only 7.3 cm^2^ V^−1^ s^−1^. Li et al. [47] use different electrolytes (0.001 M Tetraalkylammonium (TAA) salts and DMSO) and voltage (−5 V low voltage) to achieve rapid expansion and stripping of large BPS in a few minutes. The prepared BP has good size uniformity and electrical properties, with an average thickness of five layers, an average transverse area of 10 μm^2^, and a hole carrier mobility of 100 cm^2^ V^−1^ s^−1^. In addition, H_2_SO_4_ [50] and tetrabutylammonium hexafluorophosphate (TBA) [46] have also been reported as electrolytes for BP electrochemical stripping.

Therefore, high quality and uniform phosphorusene can be prepared by selecting suitable electrolyte and voltage. Electrochemical stripping opens up new possibilities for industrial production of phosphating materials.

### 2.3. Chemical Vapor Deposition (CVD) Method

The CVD method is widely used in the preparation of 2D materials. It has a bottom-up method to manufacture large size and high-quality 2D materials, including graphene, hexagonal boron nitride, transition metal dichalcogenides (TMDs), etc. [14,51,52]. Because of this, the use of the CVD method to prepare 2D materials has attracted the attention of scholars.

**Figure 2:**
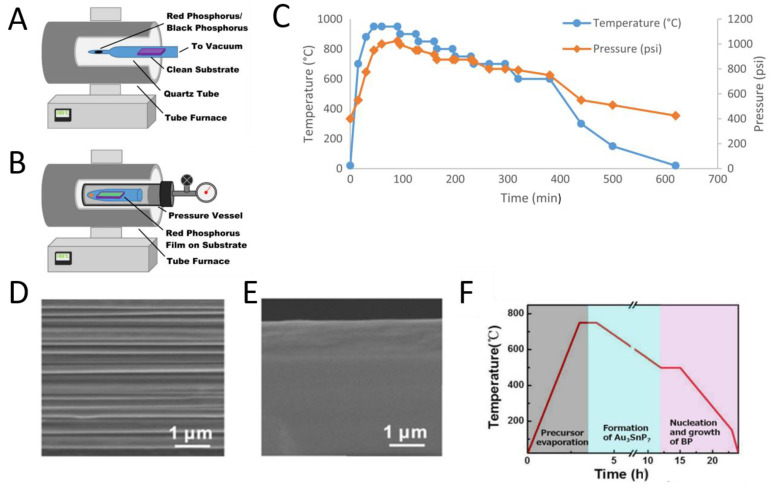
Synthesis of 2D BP nanosheets by the CVD method: (**A**) Schematic of amorphous red phosphorus thin film growth from vapor deposition of red phosphorus powder/BP piece; (**B**) Standard temperature/pressure ramp for growth of substrate BP (SBP) from the red phosphorus thin films. Temperature recorded was that of the tube furnace; (**C**) Schematic for growth of SBP on substrate from amorphous red phosphorus thin film in pressure vessel reactor [53]. (**D**) Comparison of microstructure between layered BP films grown on Si/SiO_2_ substrates and conventional compact extruded layered BP bulk crystals; (**E**) Conventional bulk BP crystals; (**F**) Diagram of experimental reaction temperature gradient [54].

In 2016, the direct synthesis of 2D BP via CVD was reported by Smith et al. [53]. The experimental device is shown in Figure 2A,B. The temperature and pressure are monitored during the experiment, and a typical ladder diagram is shown in Figure 2C. Through this method, amorphous red phosphorus was directly grown on the silicon substrate, and the thickness of the sample was about 600 nm. This work finally obtained four layers of BP film through the experiment, but also improved the safety of the experiment, and played a role in promoting the mass production of BP film. In the same year, Xu et al. [54] used the gas phase growth strategy of epitaxial nucleation design and further transverse growth control to directly grow large-size BP films on insulating silicon substrates. The maximum transverse size can reach the level of millimeters, and the thickness can be regulated from several nanometers to several hundred nanometers. The experimental temperature gradient is shown in Figure 2F. After testing and analysis, the BP films obtained have excellent electrical properties. The field-effect mobility and Hall mobility at room temperature are over 1200 cm^2^ V^−1^ s^−1^ and 1400 cm^2^ V^−1^ s^−1^, respectively, which are similar to the films stripped from BP bulk crystal materials [55]. As shown in Figure 2D,E, by comparing the microstructure of the layered BP film grown on Si/SiO_2_ substrate with that of the conventional layered BP crystal, it is found that the BP film synthesized by the substrate has a unique layered structure, which is quite different from that of the conventional BP crystal film. At the same time, the prepared BP also shows excellent optical properties in the infrared band, with enhanced infrared absorption and photoluminescence characteristics.

The gas phase growth method provides a new way for the controllable preparation of large-area and high-quality BP thin films, which is expected to be widely used in general optoelectronic devices and integrated circuits [56,57]. Liu et al. [58] achieved high quality BP growth using an efficient short distance transport (SDT) growth method with a yield of 98%. This method does not need to set temperature gradient and is a short distance transportation growth method at uniform temperature. Lange et al. [59] proposed to use the polyphosphate Au3SnP7 as the nucleation site, and realized the large-scale growth of BP film with high crystallinity on silicon and other insulating substrates for the first time.

However, there are still some problems in the process of the experiment, such as the difficult sealing of the ampoule, the formation of white phosphorus in the reaction process, and the harsh reaction conditions (not reaching the appropriate temperature and pressure will lead to the rupture of the ampoule).

### 2.4. Phase Transition and Solvothermal Reaction Method

**Figure 3:**
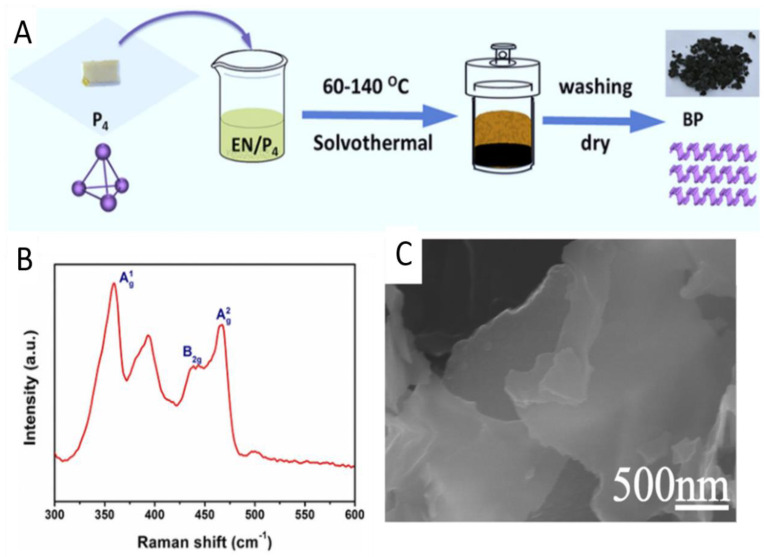
(**A**) Layer BP was synthesized from white phosphorus and ethylenediamine. (**B**) Raman spectrum. The excitation wavelength is 633 nm [60]. (**C**) SEM image of BP nanosheets synthesized by ammonium fluoride. Reproduced with permission [62]. Copyright 2016, Wiley.

In 2018, Tian et al. [60] successfully prepared small BP nanosheets using a simple, scalable, and low-cost method, the key factors of which are the choice of solvent and temperature, as shown in Figure 3A. Using white phosphorus as raw material and ethylenediamine as solvent, small layer BP was successfully prepared at 60–140 °C. As shown in Figure 3B, typical Raman peaks at 360.2, 437.5 and 464.6 cm−1 can be observed by Raman spectroscopy, corresponding to the characteristic peaks A1g, B2g, and A2g [61] of BP, which further proves the formation of BP. The solvothermal method can improve the stability of BP nanosheets, which provides conditions for rapid development and application of BP. Zhao et al. [62] used ammonium fluoride to reduce the surface activation energy of red phosphorus. Assisted by NH4F and based on the phase transformation of phosphorus and Gibbs free energy theory, a new type of 2D polycrystalline BP nanosheet was prepared for the first time by mild method, as shown in Figure 3C. NH4F can make the surface of phosphorus smooth, and in the reaction process, phosphorus is cut into small pieces by water, which plays an important role in the structure and morphology characteristics of BP cambium.

In 2016, Zhang et al. [63] demonstrated a sublimation-induced approach to prepare few-layer 2D holey phosphorus-based nanosheets from bulk RP under a wet-chemical solvothermal reaction. The mechanism of this approach includes solid–vapor–solid transformation driven by continuous vaporization condensation process, as well as sub-sequent bottom-up assembly growth.

Currently, the bottom-up method development is still at the initial stage with quite a lot of challenges to be solved. All reports related to the direct growth of thin-layer BP must start from RP, followed by phase transition.

This section summarized some classical methods of preparing 2D BP and compared their characteristics, as shown in Table 1.

## 3. Passivation of 2D BP

Compared with graphene and other traditional 2D nanomaterials, BP shows great potential in chip manufacturing, photoelectric sensing, biomedicine, and other fields because of its lamellar tunable direct band gap [63], high carrier mobility, and anisotropic thermoelectric properties [69]. In addition, BP with few layers also has excellent mechanical strength, excellent thermal stability, and high specific surface area, which makes it a potential nanofiller for preparing ideal composite materials [70].

However, in the practical application of BP, in addition to the lack of large area of high-quality sample synthesis method, its easy degradation in air is also one of the main shortcomings hindering the development of BP. Related studies [71,72] showed that the reasons for the unsteady layer BP structure are that each phosphorous has five valence electrons (3s^2^3p^3^), three electronic distribution on three 3p orbital, one lone pair electron distribution in 3p on a 3s orbital orbit of electron, and three adjacent covalent bindings of phosphorus atoms, and on the 3s orbital electrons are retained these 3s orbital lone pair electrons that have high reaction activity with oxygen and form the P_x_O_y_ group, leading to rapid degradation of BP under environmental conditions. Therefore, how to passivate the 3s orbital lone pair electrons that prevent phosphorus atom from reacting with oxygen should be the key to improve the stability of BP.

### 3.1. Ion Modification

**Figure 4:**
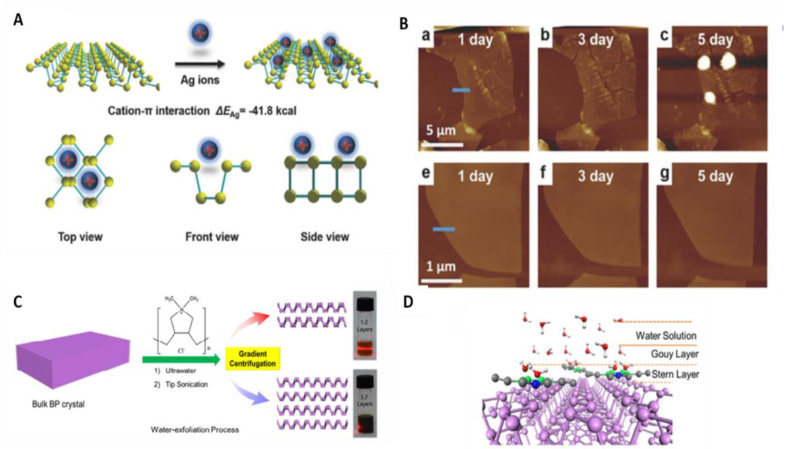
(**A**) Schematic for Ag^+^ adsorbing on BP and their three different views; (**B**) AFM images of an unmodified BP sheet (top) and Ag ^+^ —modified BP sheet (bottom) exposed to air for one day, three days, and five days. Reproduced with permission [38]. Copyright 2017, Wiley. (**C**) Schematic of the fabrication process of exfoliated few-layer BP in water by PDDA, Tyndall effect of BP colloid solution via centrifugation at 15,000 rpm (top) and 11,000 rpm (down) with bulk BP crystal (10 mg). (**D**) Schematic of adsorption process for PDDA-BP nanosheets in water. Reproduced with permission [74]. Copyright 2018, ACS Publications.

The principle of metal ion modification is to start from the direction of lone pair electrons occupying BP, so that BP lone pair electrons can be combined with metal cations, thus preventing BP from reacting with oxygen. In 2017, Guo et al. [38] calculated by density functional theory (DFT) that the binding energy of silver ion and BP was 41.8 cal, indicating that free Ag^+^ could adsorb on the surface of BP and the interaction between them was strong enough. Therefore, Ag^+^ was selected for surface modification of BP. The modification mechanism is shown in Figure 4A. The relationship between Ag+ and phosphorus atoms is one-to-many rather than one-to-one [73]. Compared with the oxidation degree of the modified BP nanosheet exposed to air (Figure 4B), it was found that the electrical performance of the BP transistor modified based on Ag^+^ was significantly improved, with a hole mobility of 1666 cm^2^ V^−1^ s^−1^ and a switching ratio of 2.6 × 10^6^, which were two and 44 times higher than that of the bare BP transistor, respectively. In addition to Ag^+^, the effects of Mg^2+^, Fe^3+^, and Hg^2+^ on crystal stability and transistor performance were also studied. The results show that different ions have different extranuclear electrons, which improve crystal stability and transistor performance in different degrees [74]. In 2018, Feng et al. [75] used Poly Dimethyl Diallyl Ammonium to carry out surface modification of BP (Figure 4C). The PDDA is selected to spontaneously and uniformly adsorb on the surface of few-layer BP via electrostatic interaction (Figure 4D). The positive charge-center of N atom of the PDDA, which passivates the lone-pair electrons of P, plays a critical role in stabilizing the BP. Meanwhile, the PDDA could serve as hydrophilic ligands to improve the dispersity of exfoliated BP in water [76]. The thinner PDDA-BP nanosheets can stabilize in both air and water even after 15 days exposure. Finally, the uniform PDDA-BP-polymer film was used as saturable absorber to realize passive mode-locking operations in a fiber laser, delivering a train of ultrafast pulses with the duration of 1.2 ps at 1557.8 nm. This work provides a new way to obtain highly stable few-layer BP, which shows great promise in ultrafast optics application [77].

**Figure 5:**
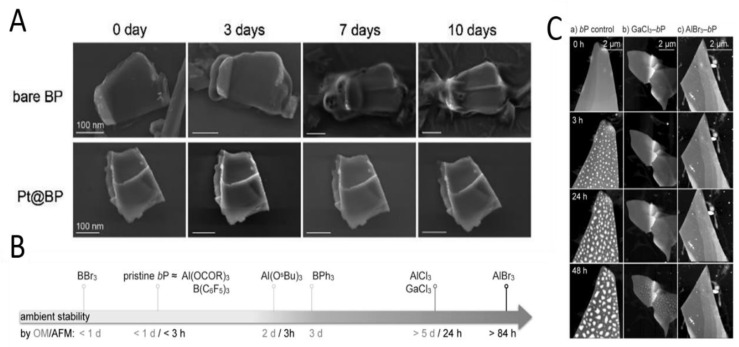
SEM images of bare BP and Pt@BP exposed in water for (**A**). Reproduced with permission [78]. Copyright 2019, ACS Publications. (**B**) Commercial group 13 Lewis acids investigated for the passivation of BP nanoflakes, ordered by their relative effectiveness according to optical and atomic force microscopy (R = C_17_H_35_); (**C**) AFM (**a**–**c**) analysis of control-BP, GaCl_3_-BP, and AlBr_3_-BP after 0 to 48 h of ambient exposure. Reproduced with permission [79]. Copyright 2021, Wiley.

In 2019, Zhang et al. [78] first tried to stabilize BP with cationic cisplatin drugs. They reacted cisplatin, oxaliplatin, and cisplatin oxidized by H_2_O_2_ with BP, respectively, and observed no significant change in binding energy. Subsequently, the two chloride ions of cisplatin were replaced by nitrate to improve the positive of cisplatin, and the binding energy of Cisplatin and BP reached 133.0 eV. After modification, the surface morphology of PT@BP did not change significantly after 24 h exposure in water and PBS. Finally, the stability of exposed BP and PT@BP in a longer period was further detected. As shown in Figure 5A, after 10 days in environmental conditions, the surface morphology of exposed BP completely disappeared, while PT@BP did not change significantly, which proved that cisplatin modification played a good role in the protection of BP. In 2021, Tofan et al. [79] developed a low-cost, high-efficiency liquid phase experimental method for surface modification of BP using 13 Group Lewis acid and demonstrated its effectiveness in environmental stability and regulation of electronic properties. Because of the electrophilic property of the reagent, Lewis acid completely inhibits the N-type conductivity [80]. The reagent can not only form Lewis adduct with BP surface, but also capture electrons at high gate voltage state. Three factors determine the effectiveness of Lewis acid in protecting BP from environmental oxidation: the electrophilicity of the Lewis acid, the Pearson hard-soft match between the acid and the phosphine, and the steric bulk of the ancillary ligands. Therefore, the use of 13 group halides (such as AlBr_3_ or GaCl_3_) can be the strongest adduct, as shown in Figure 5B. Subsequently, the samples were exposed to air, and when exposed to the environment for 3 h, BP showed visible degradation, while Lewis acid-treated BP was significantly more stable, as shown in Figure 5C.

### 3.2. Coating

**Figure 6:**
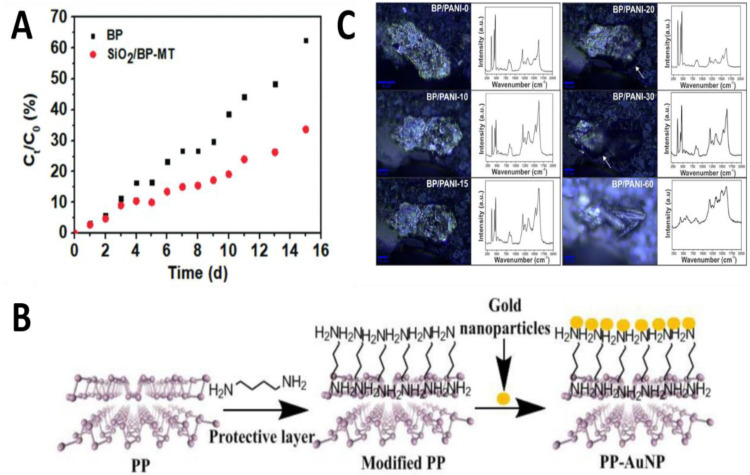
(**A**) The exposed BP and modified BP degraded over time. Reproduced with permission [87]. Copyright 2020, The Royal Society of Chemistry. (**B**) HMA is coated on BP and attached to the surface with gold for biosensors. Reproduced with permission [85]. Copyright 2018, Elsevier. (**C**) Optical images and Raman spectra (λ  =  532 nm) of BP/PANI nanocomposite deposited over a glass substrate after 0, 10, 15, 20, 30, and 60 days of exposure to ambient conditions. Reproduced with permission [82]. Copyright 2017, Springer Nature Limited.

The current modification of BP mainly involves coating its surface to prevent contact with oxygen and water in the air. In recent years, there are many kinds of coatings, such as ionic liquid [81], polymer [82], organic matter [83,84,85], and layered inorganic oxide [86,87,88,89]. Two-dimensional BP can be modified by organic matter, ionic liquid, inorganic oxide, and others, coated on the surface of BP, so that BP does not contact with oxygen and water in the air, so as to achieve the effect of BP passivation. Li et al. [87] put forward a kind of convenient, environmental protection, widely used to inactivate the BP method of nanometer sheet, 3-amino propyl-triethoxy silane, and methyl triethyl silane hydrolysis condensation on the surface of BP form hydrophobic shell and on the physical layer of BP nanosheets to prevent contact with water and oxygen, and then introduced hydrophilic silica shell. Thus, BP is hydrophobic. As shown in Figure 6A, within 15 days, the degradation degree of exposed BP reached 62.6%, while the degradation degree of BP suspension modified by SiO_2_ was only 33.7%. It can be proved that the BP modified by SiO_2_ has hydrophobicity, which can effectively shield the water on the surface of BP and slow down its degradation. Liang et al. [85] proposed passivating BP in chloroform using a self-assembled monomolecular layer of h6-methylenedi amine (HMA) to eliminate its instability in oxygen and moisture conditions, as shown in Figure 6B. HMA coating not only keeps the original honeycomb structure of BP, but also has good electrical conductivity, and enhances the stability and dispersion of BP in aqueous solutions. Its stability allows BP to remain in its original form in aqueous solutions for more than a month.

In 2017, Fonsaca et al. [82] reported a simple and effective method to modify BP by preparing conductive polymer polyaniline (PANI) nanocomposite to passivate BP, using a liquid/liquid interface method to prepare highly stable, uniform, independent polyaniline covered BP film, which has high stability in environmental conditions. It was stable in environmental conditions for 60 days, while unmodified bare BP was degraded in only three days [90]. As shown in Figure 6C, it was found by optical image and Raman characterization that BP/Polyaniline nanocomposites could be stably maintained for about 20 days under environmental conditions. After that, due to the absorption of water, some physical changes occurred on the surface of the composites, such as volume expansion and irregular surface reflection hindering the focusing of the microscope [91,92]. After 30 days, although the morphology of the thin sheet changed, its Raman characteristics remained unchanged, indicating that only part of the BP on the surface was oxidized, which also indicated that the conductive properties of the material remained unchanged during this period of time. It was not until 60 days later that the BP/Polyaniline nanocomposites lost the Raman characteristics. This modification method is highly effective for the passivation of small layers of BP, increasing the service life of BP by about 600%. In 2020, Maria Caporali et al. [93] coated BP with colloidal nickel nanoparticles, and after Raman and XPS tests, the oxidation rate of BP modified by nickel nanoparticles decreased by more than three times.

### 3.3. Doping

**Figure 7:**
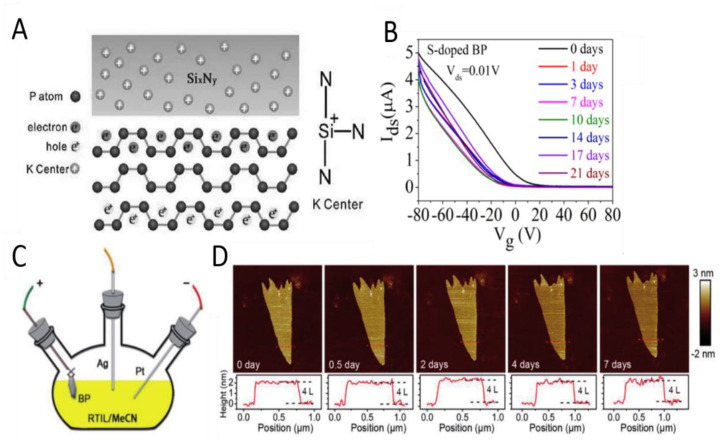
(**A**) Scheme of the field-induced n-doping of BP with Si_x_N_y_. Reproduced with permission [94]. Copyright 2017, Wiley. (**B**) Transfer curves of S-doped BP FETs at fixed drain−source voltage (V_ds_) of 0.01 V after exposure times of 0, 1, 3, 7, 10, 14, 17, and 21 days. Reproduced with permission [95]. Copyright 2018, ACS Publications. (**C**) Schematic of the experimental setup. (**D**) AFM images showing evolution of the morphology of a fresh few-layer FP under persistent exposure to ambient conditions from 0 days (no exposure) to 7 days. Height variations of the FP along the positions indicated by the red dashed lines for different exposure periods are shown below the images. Reproduced with permission [98]. Copyright 2018, Wiley.

Doping has been proved to be a simple and effective method to adjust the intrinsic properties of 2D materials. For BP, by introducing heteroatoms, the 2D structure can be adjusted to improve its physical and chemical properties, including instability, and different doping types and ways can improve the performance of BP in some respects. In 2017, Xu et al. [94] developed a stable complementary metal oxide semiconductor (COMS) compatible electron doping method, which is realized with the strong field-induced effect from the K^+^ center of the silicon nitride (Si_x_N_y_), as shown in Figure 7A. By doping Si_3_N_4_, BP remains stable for more than a month, and the electron transfer efficiency of BP can also be improved. This work provides a promising N-dope strategy for BP as well as other 2D semiconductors and paves the way toward high-performance BP-based complementary logic electronics, light-emitting diode, photovoltaic devices, and so on. Lv et al. [95] proposed a sulfur doping to inhibit the degradation of BP, and the S-doped BP field effect transistors (FETs) were more stable under environmental conditions, with carrier mobility reduced from 607 to 470 cm^2^ V^−1^ s^−1^ (still 77.4%) after 21 days of exposure in air. Environmental stability is an important characteristic of semiconductor devices [96,97]. In order to study the stability of S-doped BP FETs, the exposure time was increased, and the transmission curve was tested at a relatively stable humidity of 45–50% and temperature of 25 °C, as shown in Figure 7B. Compared with undoped BP FETs, the drain-source current I_ds_ decreases much more slowly due to the gradual increase in resistance due to degradation.

Wang et al. [99] studied the relationship between the electronic structure and magnetic properties of BP doped with Si and S by first-principle calculation. The results show that the stability of doped phosphorene could be improved by increasing the size of heteroatom or by decreasing the doping concentration. In the absence of spin polarization, the band structure of Si and S doped phosphorene always exhibits a metallic state, indicating that the band gap is not sensitive to the plane size and doping amount of the supercell. The significance of BP doping is revealed through theoretical research. On the basis of theoretical research, Zheng et al. [100] proposed a surface modification method for small layers of BP by high vacuum Al in-situ deposition, which significantly increased the electron mobility. The results show that the covalent bond is formed between BP surface layer and Al atom, and the electron mobility is greatly increased by more than six times. Because of its high electron and hole mobility, Al-doped BP can be used as high-performance logic devices or other functional electronic and optoelectronic devices. Wang et al. [101] studied the stability and electronic structure of al doped low layer BP prepared by ALD method. Al doping not only improved the stability of BP, but also improved the threshold voltage and electronic mobility of BP-based FET. Tang et al. [98] prepared large-scale F-doped phosphorusene (FP) using a simple one-step ionic liquid-assisted electrochemical stripping route. The experimental device is shown in Figure 7C. FP inherits the anti-oxidation and anti-hydration properties of fluorine atoms with high electronegativity, so that it has air-stable photothermal properties and can be stored for more than a week, as shown in Figure 7D.

This section summarized some passivation methods and their passivation effects of 2D BP in recent years, as shown in Table 2.

## 4. Applications of 2D BP based LIBs

LIBs work by converting electrical and chemical energy, or charging and discharging, between their anode and cathode [103,104,105,106]. Therefore, in theory, as long as the chemical structure of positive and negative active materials of LIBs is stable and the conversion cycle of electric energy and chemical energy is carried out, the battery can be used for a long time. The performance of LIBs mainly depends on the stability, conductivity, and conversion rate of anode and cathode materials [107,108,109,110]. At present, the cathode materials of LIBs are mainly lithium iron phosphate [111], nickel cobalt manganese ternary compound [112], and the cathode materials are mainly graphite and carbon [113,114]. Anode materials, especially active parts, play an important role in LIBs, so they are now the main focus. It is mainly because of the key technological breakthrough of LIBs anode material, which can greatly improve the energy density and power density of LIBs, and that plays an extremely important role in the performance of LIBs. Lithium example batteries typically consist of an anode, a cathode, and an electrolyte, where the charge flow is generated by the intercalation and delamination of lithium-ions between the anode and cathode. As is known to all, the selection of electrode materials plays an important role in the performance of LIBs.

The theoretical capacity of BP is 2596 mAh g^−1^, which provides the basis for the capacity of LIBs. At the same time, BP has good electrical conductivity, which provides the possibility for rapid charge and discharge preparation of high-rate LIBs. These characteristics show great potential in the preparation of high-performance LIBs. On the other hand, BP’s unique fold structure provides more space for the insertion of Li^+^, and Li atoms can combine with P atoms to form strong bonds. Qiu et al. [115] believed that this process can be divided into BP-LiP-Li_2_P-Li_3_p, which provided a low diffusion energy barrier (0.08 eV) for the mobility of Li atoms [116,117]. Zhang et al. [118] calculated theoretically that the diffusion mobility of Li^+^ in BP along ZZ direction was about 10^7^–10^11^ times that of AC direction. This directional diffusion mobility is far superior to other 2D materials such as graphene and MoS_2_, which allows BP to have ultra-fast charge/discharge characteristics. In addition, the 2D structure of BP has high reversibility in the process of lithium intercalation. In other words, during the charging and discharging process, the volume change of monolayer BP is only 0.2%, while that of bulk BP is 300% [119,120]. The thin layer BP becomes one of the most promising candidate materials for LIBs in the future due to its large storage space, extremely fast diffusion rate of Li^+^, and stable and reversible structure.

On this basis, the experimental study of BP as anode material of LIBs was carried out. Castillo et al. [121] prepared a small layer BP with an average transverse size of 30 nm and an average thickness of 13 layers by liquid phase stripping method as the anode of LIBs. At the current density of 100 mA g^−1^, the electrochemical performance of the prepared BP was tested with a half cell. The initial capacity was 1732 mAh g^−1^. However, the electrode showed large capacity fading in the first 10 cycles. After 100 charge and discharge cycles, the specific capacity attenuates to 480 mAh g^−1^. Zhang et al. [122] also prepared several layers of BP (5–12 layers) by liquid phase spalling method. However, the BP electrode with few layers exhibits the same rapid capacitance decay, resulting in a coulomb efficiency (CE) of only 11.4% and a reversible specific capacity of only 210 mAh g^−1^, which are not satisfactory. They suggest that these poor properties may be due to the thicker layers of BP, resulting in large volume changes during the cycle. Therefore, many scholars proposed the construction of BP composite materials to overcome the volume expansion, low coulomb efficiency, and low reversible capacity of BP in LIBs.

### 4.1. BP/C Composite Materials

In 2014, Sun et al. [123] found that four different BP/C composites were prepared by high-energy mechanical milling using different carbon sources (graphite, graphite oxide, carbon black, fullerene). The effects of four different P-C bonds on chemical properties were studied.

**Figure 8:**
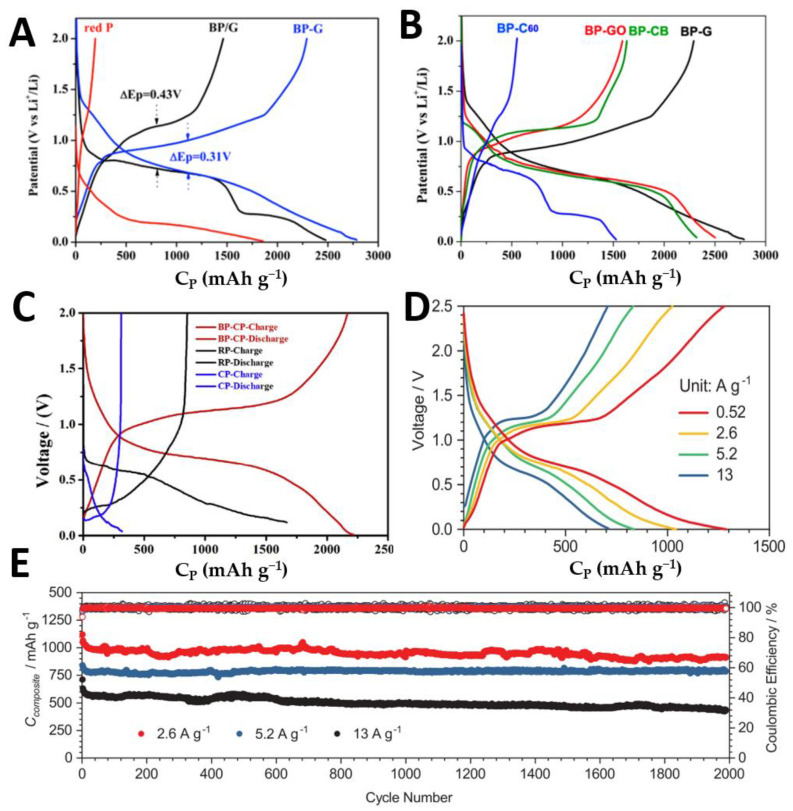
(**A**) First charge–discharge curves of Red P, BP/G, and BP-G at 0.01–2.0 V current density. (**B**) First charge–discharge curves of BP-C60, BP-GO, BP-CB, and BP-G at 0.01–2.0 V current density. Reproduced with permission [123]. Copyright 2014, ACS Publications. (**C**) The charge–discharge profiles of the initial cycle in the voltage range of 0–2 V. Reproduced with permission [124]. Copyright 2018, Elsevier. (**D**) Constant current charge and discharge and charge distribution under different current densities. (**E**) Cyclic performance of BP-G /PANI at current densities of 2.6, 5.2, and 13 A g^−1^. Reproduced with permission [125]. Copyright 2020, Springer Nature Limited.

The coherent P-C bond formed in BP-G composite material provides high capacity and good cycling capability for battery performance. Figure 8A evaluates the electrochemical lithium storage properties of RP, BP, and graphite mixtures (BP/G) without high energy mechanical milling, and BP-G composites chemically bonded by high energy mechanical milling by constant current charge–discharge measurements. CP and CP/G are the specific capacities calculated according to the weight of BP and BP-G composite materials, respectively. During the lithium process, when the potential drops from the initial 2V to 0.78, 0.63, and 0 V. The multistep reaction is attributed to the transformation of BP-LiP-Li_2_P-Li_3_P. Despite the initial CP charge of BP/G capacity electrode of 2479 mAh g^−1^, the coulomb efficiency caused by the first cycle was only 58% resulting in the loss of electrical contact with G. BP and G (BP-G) through P-C bond, when circulating in the voltage range of 0.01 and 2.0 V, has an initial CP specific capacity of 2786 mAh g^−1^. The reversible CP specific capacity is about 2382 mAh g^−1^. The hysteresis (δ Ep) of the discharge and charging platform is reduced from 0.43 V to 0.31 V. The decrease of δ Ep indicates that PC bond improves coulomb efficiency due to better connection between particles in the process of delithium and lithium embedding with large volume variation. As shown in Figure 8B, in the constant current charge–discharge curve, it can be seen that BP-G has the highest specific capacity compared with four different P-C bonds. In 2017, Jiang et al. [124], for the first time, directly synthesized BP on conductive carbon paper (BP-CP) by efficient thermal evaporation deposition method to form black phospho-carbon composite material (BP-CP), which was successfully used as the anode material of LIBs, and evaluated the electrochemical lithium storage performance of RP, BP-CP, and bare CP by constant current charge–discharge measurement (see Figure 8C). The discharge/charge capacities of bare CP and RP are 31.9/318.9 and 1668.7/852.4 mAh g^−1^, respectively, which are too small to be used as anode materials for LIBs. From the semiconductor point of view, BP has a low genetic conductivity. In order to improve the electrochemical performance of BP, BP samples grown directly on carbon paper may have better conductivity, which can promote the electrochemical behavior of BP in the discharge/charging reaction with lithium-ions. Compared with bare CP and RP, the charge and discharge capacities of BP-CP at 0.1 C current density increased to 2219.1 and 2168.8 mAh g^−1^, respectively (Figure 8C).

In 2020, Jin et al. [125] developed the preparation of polyaniline coated BP-graphite by in-situ polymerization of BP as an active anode for high-rate and high-capacity LIBs by ball milling a mixture of polyaniline and graphite. The formation of covalent bonds with graphite and carbon inhibits the boundary reconstruction of layered BP particles and ensures the rapid entry of Li^+^ into the open edge. The covalently bonded BP-graphite particles were coated with expanded polyaniline to form a stable solid electrolyte interface phase, which inhibited the continuous growth of lithium fluoride and carbonate with poor conductivity, and greatly reduced the volume expansion problem of BP when used in LIBs. The expanded polyaniline induced the doping of Li^+^ and proton in the polymer matrix, absorbing HYDROGEN fluoride and promoting the charge transfer between electrode and electrode electrolyte interface. According to the reversible capacity test at different current densities, the reversible capacity can reach 1300 mAh g^−1^ at 0.52 A g^−1^, as shown in Figure 8D. Reversible capacity of 440 mAh g^−1^ at high current density of 13 A g^−1^ in 2000 cycles (Figure 8E). When the load mass is 3.6 mg cm^−2^, the capacity is maintained at 750 mAh g^−1^ at 0.52 A g^−1^.

### 4.2. Other BP Composite Materials

**Figure 9:**
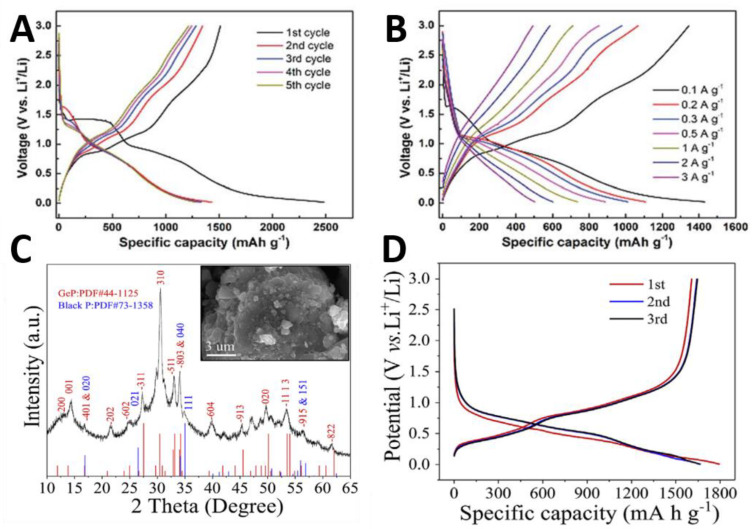
(**A**) Discharge/charge profiles of the first five cycles at 0.1 A g^−1^. (**B**) Discharge/charge profiles at various rates. Reproduced with permission [126]. Copyright 2019, The Royal Society of Chemistry. (**C**) The XRD pattern and the inset shows the morphology. (**D**) The initial three discharge/charge profiles of the mixture of 2Ge and 3P milled at 7 h. Reproduced with permission [127]. Copyright 2019, Elsevier.

Due to the large volume change and low cycle life of BP when it is used as the anode of LIBs, Jin et al. [126] designed and prepared a BP nickel-cobalt alloy composite (BP/NiCo MOF), which is composed of Ni^2+^ and Co^2+^ in benzenedicarboxylic acid (BDC). The hydroxyl group in BDC^2-^ can not only chelate with metal ions, but also bond with BP to form a stable 2D hybrid structure. BP/NiCo MOF electrode can provide abundant redox active sites to ensure the lithium storage capacity of the electrode. The nanostructures and porous structures after two days in ambient conditions not only retained a good charge transfer, but also buffered multi-loop volume expansion, so that the materials have good cyclic stability and excellent velocity performance, and ultimately improved the electrochemical performance. At the rate of 0.1 A g^−1^, the capacity of the first cycle charge and discharge can reach 2483 mAh g^−1^. After five cycles, the capacity of the cycle charge and discharge still remains at 1512 mAh g^−1^, as shown in Figure 9A. After analysis, the irreversible capacity loss is due to the decomposition of electrolyte to form solid electrolyte interface (SEI) and the partial capture of Li^+^ at the active site of hybrid structure. As shown in Figure 9B, by comparing the charge–discharge curves under different current densities, its cyclic charge–discharge capacity can still reach nearly 500 mAh g^−1^ under the condition of high current density 3 A g^−1^. The composite material has high stability, a long cycle life, and good rate performance due to the synergistic effect of low layer BP and binary nickel-cobalt alloy structure. It provides a new way for the design and engineering of high-performance lithium storage systems.

In 2019, Li et al. [127] designed and synthesized Ge_2_P_3_ composite material based on GeP_5_’s characteristics of metal conductivity, layered structure, large capacity, and high initial coulomb efficiency in the application of LIBs anode material, through the ball milling method to make a layered GeP and BP composite. The results showed that the layered GeP-BP composite has good lithium storage ability. It can be seen from Figure 9C that Ge_2_P_3_ powder is composed of layered GeP (JCPDS-44-1125) and layered P (JCPDS-73-1358). Due to the layered crystal structure, binary reaction components and rich heterogeneous interfaces of the composite electrode, the composite electrode has high performance of lithium storage. Figure 9D shows the three initial charge and discharge efficiencies of the layered GEP-BP composite electrode, which are 1795 mAh g^−1^ and 1610 mAh g^−1^, respectively, and the initial coulomb efficiency is up to 89%. Three cyclic voltammetry curves show that the layered GEP-BP composite electrode has undergone three stages of lithium storage process: intercalation, conversion, and alloy [128].

## 5. Conclusions and Prospect

Since the successful preparation of 2D BP by the high-pressure method for the first time, researchers have made a lot of new progress in the structure, preparation and, reaction mechanism of 2D BP, especially as regards the emergence of catalytic method, which opened a new chapter in the preparation of 2D BP. However, in general, the following serious and urgent problems in the preparation of BP by catalytic method still remain: (1) The preparation of BP by the catalytic method is still an intermittent operation, which is not conducive to the large-scale preparation of 2D BP; (2) There are few studies on the preparation of 2D BP from cheap white phosphorus, which is not conducive to further reducing the preparation cost of 2D BP; (3) The formation mechanism of 2D BP is not clear, and the specific structure of the intermediate transition state during the conversion of red phosphorus to BP and the process of transition state to BP still need to be explored. To sum up, the preparation of BP with low-cost white phosphorus as raw material, in-depth study of the formation mechanism of 2D BP, and the realization of high-quality, low-cost, and large-scale preparation of 2D BP are the research directions that need to be followed in the future. In fact, the ultimate purpose of preparing BP is application, and the premise of application lies in the high quality and efficient preparation of nano-BP and its composite materials. In summary, ultrasonic stripping and electrochemical stripping are the two most commonly used methods for the preparation of nano-BP, and even effective means for the preparation of nano-BP matrix composites. However, both of these two methods have the problem of “uncontrollable” preparation of nano-BP, mainly that the size and thickness are not easy to control, which affects the application of nano-BP to a certain extent. Therefore, research on controllable preparation of nano-BP is of great significance to the application of nano-BP. Compared with the top-down method, the bottom-up solvothermal method and chemical vapor deposition method can produce nano-BP or even nano-BP matrix composites in one step. The preparation process is simple and efficient, especially the chemical vapor deposition method, which has the potential to prepare high quality nano-BP matrix composites. However, due to the immaturity of the preparation system and technology, many problems in the preparation process have not been solved, and industrial production is challenging to some extent.

At present, the coulombic efficiency, cycle performance, and rate performance of the battery have great room for improvement when nano-BP matrix composites are applied to the negative electrode of energy storage battery, and the construction of the composite material is particularly important. It is of great significance to promote the application of nano-BP in the field of energy storage using existing preparation methods or developing new methods to compound nanometer BP with existing materials or synthesis or finding new materials to construct nanometer BP matrix composites with excellent energy storage performance and develop efficient preparation methods. In addition, the theoretical calculation shows that black phosphorene also shows excellent hydrogen storage performance after metal doping. Putting the theory into practice will be an urgent research work in the next step. In summary, excellent performance depends on the structure–activity relationship between materials, and many challenges remain.

## Figures and Tables

**Table 1:** Comparison of some classical preparation methods and characteristics of 2D BP.

Method	Experiment	Thickness	Characteristic	Ref.
Chemical vapor deposition	—	~4 layers	Flexible size, a variety of lateral size samples	[53,54,58]
Pulsed laser deposition	—	2–8 nm	Flexible size with limited carrier mobility due to disordered structure	[64]
Mechanical exfoliation	Sticky-tape	<7.5 nm	High carrier mobility with low production yield limit	[65,66]
	Metal-assisted	~4 nm	Long time consuming, low yield, uncontrolled size	[34]
Sonication liquid-phase exfoliation	Sonic exfoliated in NMP	3–5 layers	A 200 × 200 nm^2^ lateral dimension	[39]
	Sonic exfoliated in formamide	~3 layers	A 50–300 nm lateral dimension, 38% yield	[67]
Electrochemical exfoliation	Two electrode system (Pt and bulk BP)	3–15 layers	A 0.5 to 30 µm lateral dimension, yield excess 80%, non-uniform size	[28,68]
	Electrolyte TAA and −5 V low voltage	~5 layers	Fine dimensional uniformity and electrical properties, average cross area of 10 μm^2^	[47]
Solvent hot method	White phosphorus as raw material, ethylenediamine as solvent	—	The synthesis is stable, and the transverse size is 0.8–1.0 μm	[60]
	Red phosphorus as raw material, ammonium fluoride as solvent	~2 layers	Large scale preparation, low cost	[62]

**Table 2:** Passivation method and effect comparison of 2D BP.

Passivation Technique	Coating Method	Oxidation Time	Ref.
10 nm Al_2_O_3_ thin film	ALD	>90 days	[102]
Ag^+^ adsorption	The few layers of BP were transferred to Si/SiO_2_ wafers with PDMS film as medium, and placed into the mixed solvent of NMP and AgNO_3_	>5 days	[38]
PDDA hydrophilic ligand adsorption	PDDA and BP spontaneously and uniformly adsorbed by electrostatic action	>15 days	[73]
Cationic cisplatin	Reacted cisplatin, oxaliplatin, and cisplatin oxidized by H_2_O_2_ with BP	>10 days	[78]
Group 13 Lewis acids	Under inert gas, the few layers of BP were stripped onto Si/SiO_2_ wafers and adsorbed with group 13 Lewis acid	>5 days	[79]
3-aminopropyl triethoxysilane and methyl triethyl silane	BP nanosheets were hydrolyzed and condensed with 3-aminopropyl triethoxysilane and methyl triethyl silane to form hydrophobic shells	>15 days	[87]
Hexamethylene-diamine	HMA monomolecular layer passivation of BP in chloroform	>30 days	[85]
PANI nanocomposites	A highly stable and uniform PANI covered BP film was prepared by liquid/liquid interface method	>20 days	[82]
Complementary metal oxide semiconductor (CMOS)	A strong field effect produced by the K^+^ center of silicon nitride (Si_x_N_y_)	>30 days	[94]
Sulfur doping	BP was synthesized under high temperature and high pressure, and then mixed with 1wt% sulfur	>21 days	[95]
Fluoride ionic liquid	Electrochemical stripping and synchronous fluorination	>7 days	[98]

## Data Availability

The data presented in this study are available on request from the corresponding author.
